# A protocol for developing a classification system of mosquitoes using transfer learning

**DOI:** 10.1016/j.mex.2022.101947

**Published:** 2022-11-28

**Authors:** Pradeep Isawasan, Zetty Ilham Abdullah, Song-Quan Ong, Khairulliza Ahmad Salleh

**Affiliations:** a Faculty of Computer and Mathematical Sciences, Universiti Teknologi MARA, Perak Branch, Tapah Campus, 32610, Perak; b Institute for Tropical Biology and Conservation, Universiti Malaysia Sabah, Jalan UMS, 88400 Kota Kinabalu, Sabah

**Keywords:** Aedes aegypti, Aedes albopictus, Deep learning, Expert system

## Abstract

Mosquito identification and classification are the most important steps in a surveillance program of mosquito-borne diseases. With conventional approach of data collection, the process of sorting and classification are laborious and time-consuming. The advancement of computer vision with transfer learning provides excellent alternative to the challenge. Transfer learning is a type of machine learning that is viable and durable in image classification with limited training images. This protocol aims to develop step-by-step procedure in developing a classification system with transfer learning algorithm for mosquito, we demonstrate the protocol to classify two species of *Aedes* mosquito - *Aedes aegypti* L. and *Aedes albopitus* L, but user can adopt the protocol for higher number of species classification. We demonstrated the way of start from the scratch, fine-tuning two pre-trained model performance by using different combination of hyperparameters – batch size and learning rate, and explain the terminology in the Appendix. This protocol target on the domain expert such as entomologist and public health practices to develop their own model to solve the task of mosquito/insect classification.

## Graphical Abstract

**Figure:**
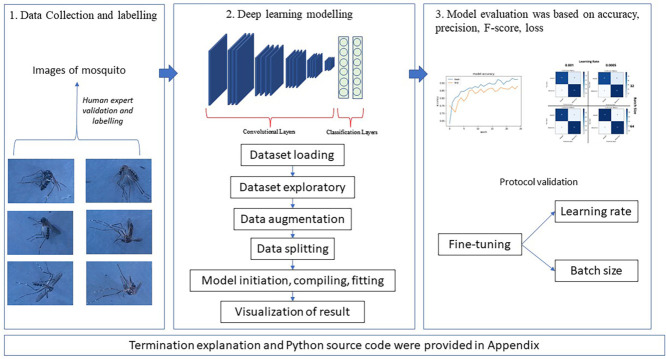

Specifications Table

**Table:** 

Subject Area:	Bioinformatics
More specific subject area:	*Deep learning, automated recognition system*
Protocol name:	*Mosquito automated recognition system*
Experimental design:	This study aims to develop a protocol for entomologist or taxonomist to build their own transfer learning model. The tool that we used to demonstrate the protocol is Google Colaboratory (Google Colab) which is using Python programming language and allows us to train a robust deep learning (including transfer learning) without the restriction of hardware, especially GPU. It also relieved us from the restriction of package or libraries installation. Overall experiment design of this protocol started with dataset retrieving, following with the process of pre-processing, data augmentation, transfer learning model training and evaluation. To validate the protocol, we trained two model with the step of fine-tuning and compare their performance in brief.
Trial registration:	Not applicable
Ethics:	Not applicable
Value of the Protocol:	• A general three-step protocol for developing a transfer learning model to classify mosquito images is offered • A description of the terminology and rationale of the model performance is offered. • Users can simulate or customize their model in Python to run through the protocol.

## Description of protocol

This study aims to develop a protocol for entomologist or taxonomist to build their own transfer learning model. The protocol is arranged according to the implementation environment, overall process flow, dataset and data pre-processing preparation, and model built-up. Each of the section is attached with the respective Appendix that consists of Python code that allow entomologist/taxonomist to implement in the Google Colab environment. The Appendix consists of a brief introduction of each method, justification of its implementation, and how it is implemented in the project's coding. Although the protocol demonstrated a classification task in distinguishing two species of *Aedes* mosquitoes, the protocol can be reused in a task with more than two classes of insects, which required some minor changes to the organisation of the folders used for inclusion in the model. An example of applying the protocol to five species of dipterous fly was shown in [1].

### Programming tools

The tool that we used is the Google Colaboratory (Google Colab) which is using Python programming language. This tool allows us to train a robust deep learning (including transfer learning) without the restriction of hardware, especially GPU. It also relieved us from the restriction of package or libraries installation.

Overall Process Flow of Transfer Learning Model Development.

**Fig. 1:**
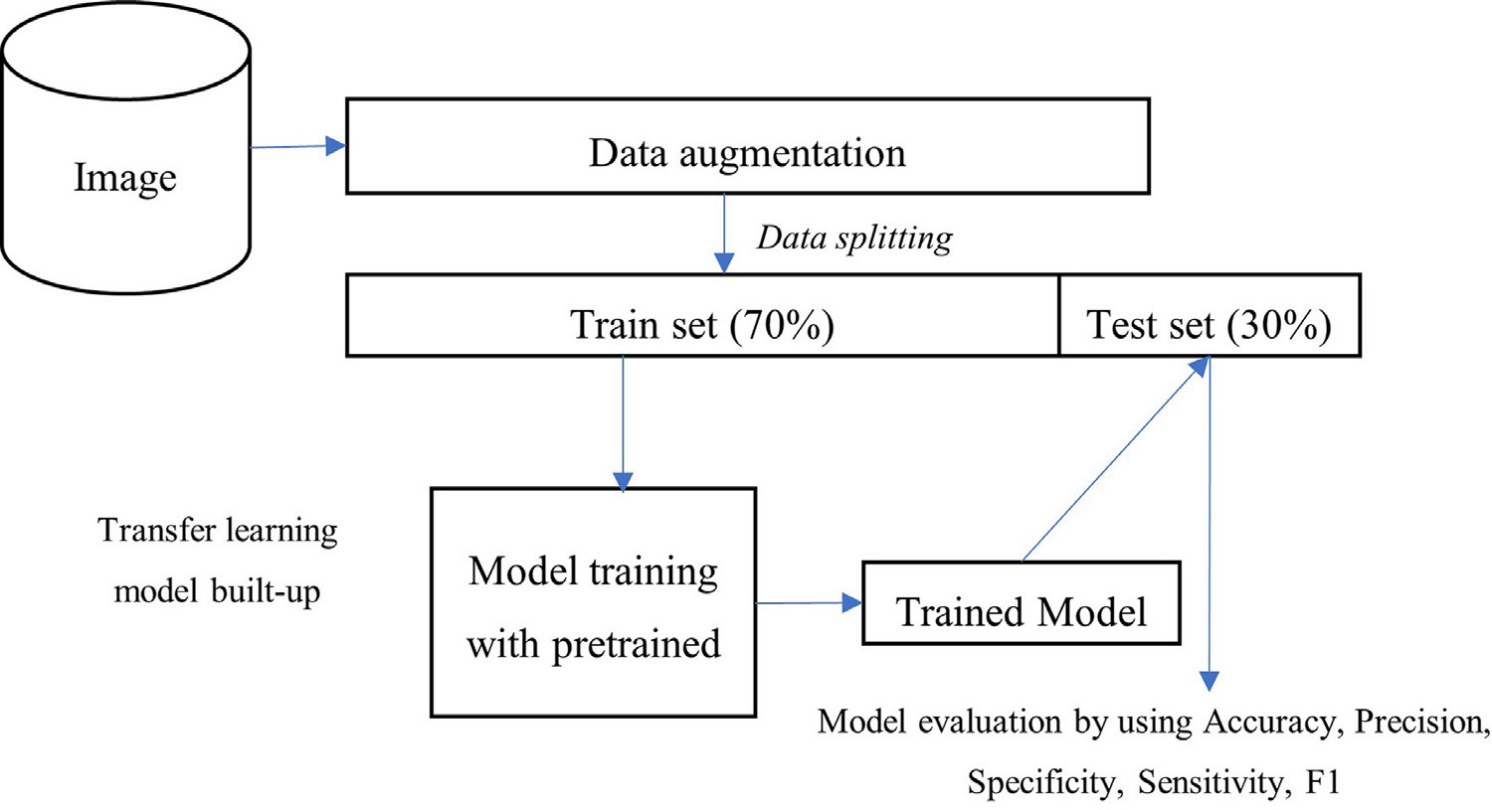
Overall process flow for developing a transfer learning model.

Fig. 1 shows the process flow of constructing a transfer learning algorithm. Using the dataset obtained, it goes through the process of pre-processing, data augmentation and train modelling. The trained model then implements transfer learning and goes through testing and validation.

### Dataset

Dataset of *Aedes aegypti* L. and *Aedes albopictus* L. are retrieved from Kaggle database [2]. The dataset has a total number of 4,800 images of *Aedes* mosquitoes, 2,400 images of *Ae. aegypti* and 2,400 images of *Ae. albopictus* respectively. It consists of images of *Ae. aegypti* and *Ae. albopictus* that are older than 12 days and the white scales on the thorax that are no longer present. A more detailed explanation can be found in Ong et al. [3] that have implemented deep convolutional neuron network (DCNN) to classify the images.

### Data augmentation and data splitting

Since the image dataset obtained from Kaggle consists of 1,205 images which may not be enough to train a robust image classification model, therefore, we proceeded with data augmentation to increase the number of images for training [4]. Data augmentation was performed on the train and test sets, which geometric transformations was done on the training data by rotating the images by 0, 90, 180 and 270 degrees. Developing a machine learning model (including the transfer learning), requires partition or splitting the data into two “set” – 1) training set, that serves as the data for the algorithm and architecture to “learn” the features and patterns form the images; 2) test set, that serves as the data to evaluate the model performance. In this study, the dataset was split into 70% train set and 30% test set (Fig. 1). The details of implementation of data augmentation and splitting were demonstrated in Appendix A, which used ImageDataGenerator package that is imported along with Keras and Tensorflow libraries.

### Transfer learning model construction

The transfer learning in this study is a type of Convolutional Neural Network (CNN) that is commonly used to train classification model. We utilized Keras API and TensorFlow libraries to retrieve two pre-trained models – MobileNetV2 and VGG16 and fine-tune the hyperparameter of the model with two levels of batch size (32 and 64), and two levels of learning rate (0.0005 and 0.001) for each batch size, respectively. Appendix A consists of the explanation for each of the terms of hyperparameter. The details of implementation of model built-up were demonstrated in Appendix B. The model evaluation was based of the matrices of accuracy, precision, specificity, sensitivity, and F1 score. The evaluation matrix was derived from the confusion matrix that have been generated from the model prediction as described in Appendix B.

### Protocol validation

**Fig. 2:**
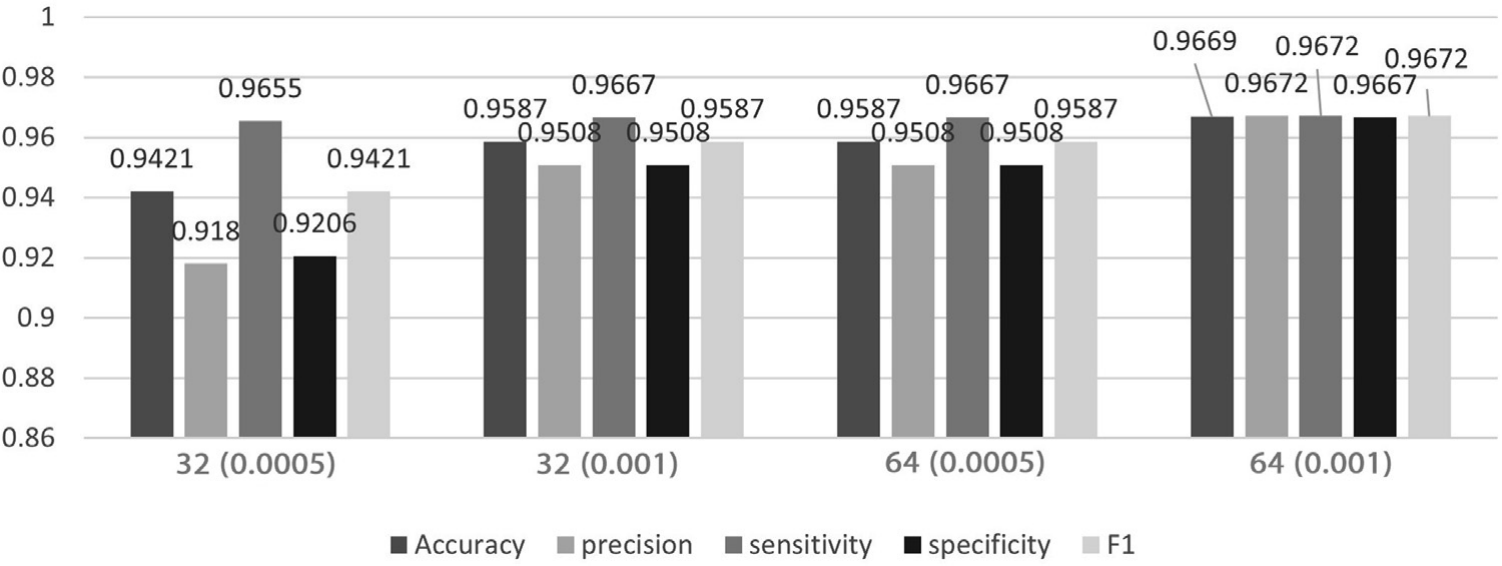
Model performance of MobileNetV2.

**Fig. 3:**
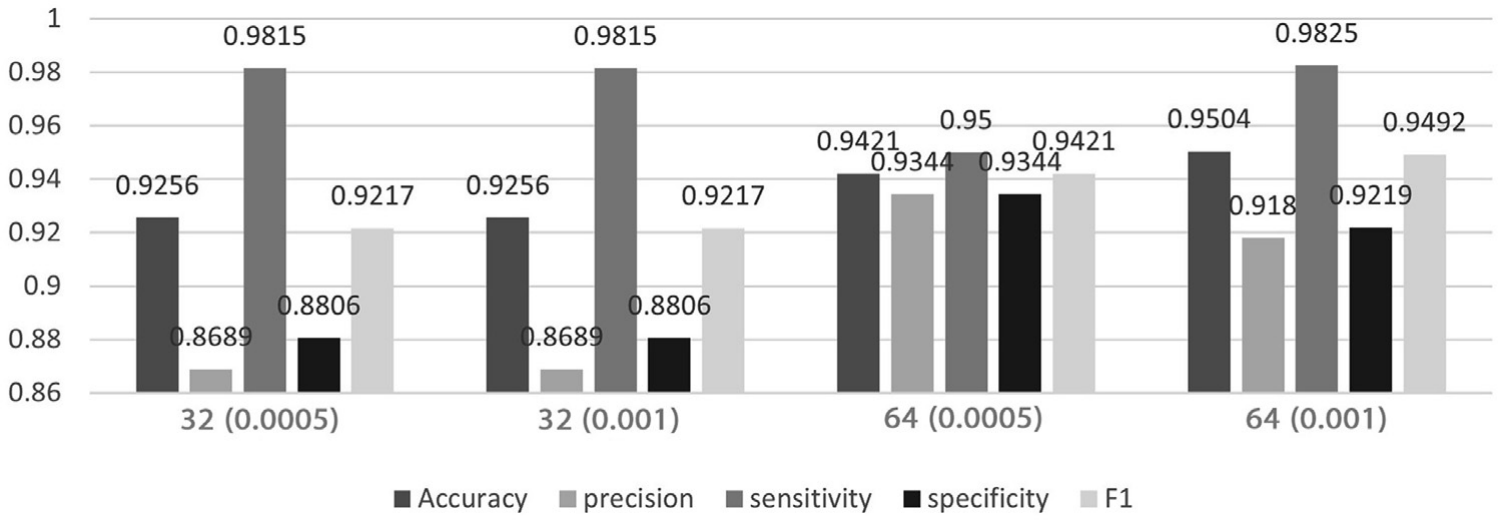
Model performance of VGG16.

To validate the protocol, we run the Python code to develop two pre-trained model – MobileNetV2 and VGG16, and Figs. 2 and 3 show the results of model performance, respectively. In general, higher batch size-64 performed greater than batch size-32. To be more specific, MobileNetV2 performed better than VGG16 with learning rate of 0.001, the model can achieve at least 0.96 for all the evaluation matrices. In terms of sensitivity, VGG16 has at least 0.98 for all the batch sizes and learning rate, except for batch size 64 with 0.0005 learning rate. Sensitivity or true positive rate that measures the proportion of actual positive (true value) are correctly predicted, which indicate the correctness of VGG16 in predicting correct classes of Aedes mosquito.

**Fig. 4:**
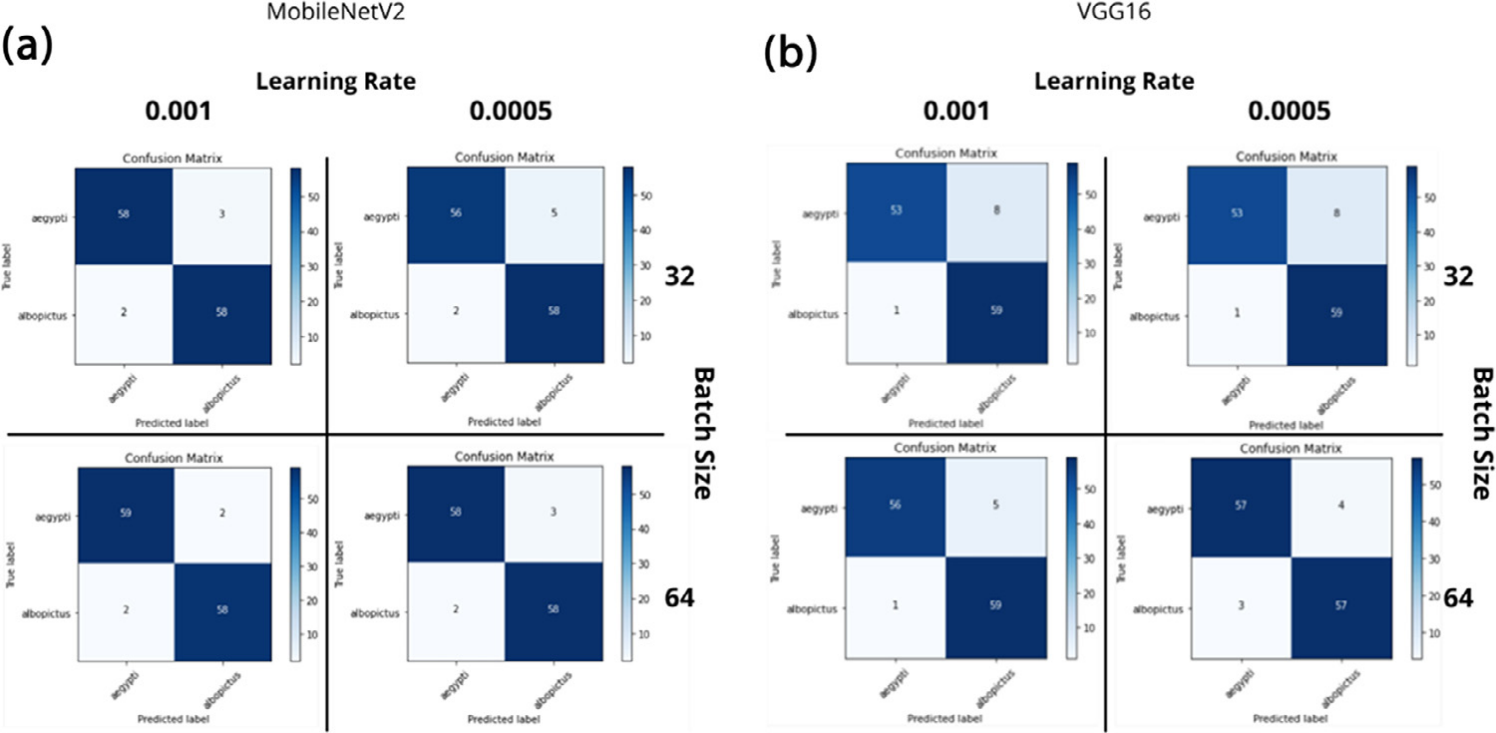
Confusion Matrix. (a) MobileNetV2 for transfer learning on four different combinations of hyperparameters; (b) VGG16 for transfer learning on four different combinations of hyperparameters.

**Fig. 5:**
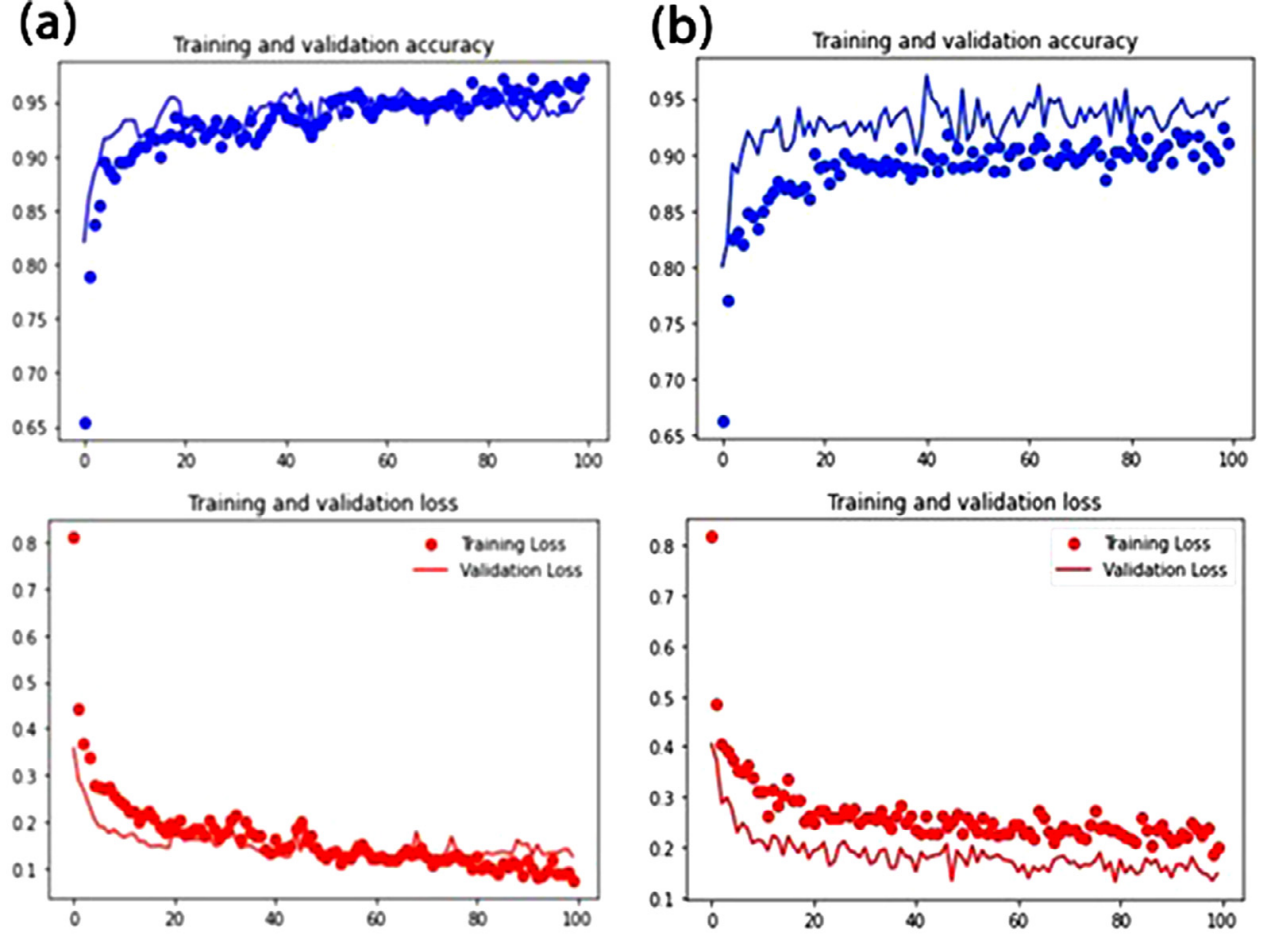
Training and testing (validation) process. (a) MobileNetV2 (b) VGG16

Fig. 4 shows the confusion matrix for MobileNetV2 and VGG16, respectively. Con-fusion matrix is a common evaluation tools used in the domain of machine learning to visualize the information of model performance such as accuracy, precision, etc. by referring to the intensity of the colour used by the matrices. For training and testing (validation) process, MobileNetV2 consists of more stable and consistent output of accuracy and loss (Fig. 5a), in relative to VGG16 (Fig. 5b).

This study aims to act as a protocol for entomologist or taxonomist to construct their own machine learning model. Therefore, the design of the architecture was constructed to be fundamental yet can be benchmarked with the latest studies. We also provided the explanation and the programming codes details and terminologies that are in-volved in processing the image data, training the transfer learning, and model evaluation in Appendix. From our result, the higher batch size-64 obtained greater result than lower batch size-32. The result obtained agrees with the studies of Smith et al [5] and Kandel et al [6] that have demonstrated how higher batch size was used to improve the generalization of a model. However, as suggested by Kevin [7], when the batch size increased until some extent, the generalization will decay, therefore, more levels of batch size could be studied to obtain the optimum result.

Most of previous studies focused on using VGG architecture such as VGG16 and VGG19 to develop the mosquito classification system [8], [9], [10], [11] due to the architecture being used to win the ILSVR and is still powerful in computer vision model till date. Relatively fewer studies on mosquito classification focused on MobileNetV2 due to its lower performance in ILSVR, and our result demonstrated that the MobileNetV2 outperformed over VGG16 at batch size-64. These findings were parallel with Ong et al [1,3] that used a platform – Teachable Machine by Google, that implement MobileNet architecture. MobileNetV2 is comparably a thinner and more lightweight network due to it having fewer parameters as compared to VGG16, which is suitable for experiments with limited training samples [12]. Theoretically, any deep CNN architecture can be used in this approach, this is proven by how both models performs with not much difference in terms of accuracy giving off more than 90% accuracy score in model testing. The application of fine tuning in transfer learning concept is proven to improve the performance of the model [13]. Additionally, despite MobileNetV2 highest accuracy score outperforms VGG16, there is a noticeable similarity that contributes as a major factor to the highest accuracy of both models. In addition, from the point of view of time and cost, MobileNet2 is relatively faster in deriving unseen data and has a small size that incurs low computational costs (Table 1). Both model MobileNetV2 and VGG16 obtained its highest accuracy score by using batch size of 32 and learning rate of 0.001 and this demonstrated a significant factor. Based on the results, to achieve the best performing model, it can be concluded and proven that hyperparameter tuning is very crucial in order to obtain high accuracies. Hyperparameters specifically the learning rate and its batch size has to carefully be decided, since it majorly affects the performance of the model.

**Table 1:** Example of transfer learning model with their respective size and time per inference step [14].

Model	Size (MB)	Time (ms) per inference step (GPU)

ResNet50	98	4.6
Xception	88	8.1
InceptionV3	92	6.9
MobileNetV2	14	3.8
VGG16	528	4.2

We have also demonstrated that the classification system of *Ae aegypti* and *Ae albopictus* can achieved at least 0.90 accuracy, precision, specificity, sensitivity and F1 score. However, it has to be emphasized that the dataset used in this project were the images taken by a specific device, with the same background, and under the same lighting, which makes the appearance of the mosquitoes’ physical features in the im-ages consistent. In real life application, if the model is to be deployed as a mobile ap-plication, it might affect the performance due to the inconsistency of certain environment such as the difference in level of brightness and colours of the background where the mosquito picture is taken. Additionally, the model architecture of this project was trained only on two different species of *Aedes* mosquitoes which are *Ae aegypti* and *Ae albopictus*. The model is limited to recognizing only the two said species which means it is not familiar with other mosquitoes’ species that are present. This will be a constraint on the model's ability to predict other mosquito species correctly. For future work, the suggestion is to expand the collection of vectors across the world taken using different devices, with various backgrounds and settings. The larger the dataset, the better the model will learn and perform in the future, thus making it possible for it to be deployed as a system and be very useful for entomologists, health workers, or even non-experts.

## Declaration of interests

The authors declare that they have no known competing financial interests or personal relationships that could have appeared to influence the work reported in this paper.

## Data Availability

I have share the link to my data/code at the attach file step
